# Supplementary material for: Finding multiple reaction pathways via global optimization of action
Source: Nat Commun. 2017 May 26;8:15443. doi: 10.1038/ncomms15443 (PMC5458546; doi:10.1038/ncomms15443)
Supplement: Supplementary Information — Supplementary Tables and Supplementary Figure [file ncomms15443-s1.pdf]

Supplementary Table 1. Parameters for Action-CSA calculations

INDEX	DESCRIPTION OF PARAMETER
**1**	Total transition time
**2**	Number of pathway steps
**3**	Number of pathways retained in bank at each iteration
**4**	Number of crossover and mutation operations
**5**	Temperature
**6**	Friction coefficient
**7**	Weight of total energy conservation term
**8**	Annealing schedule of pathway separation distance
**9**	Solvation model
**10**	Protein force field

Supplementary Table 2. List of 14 unique pathway types and 44 non-redundant pathways for conformational change of hexane from g-g-g- to g+g+g+

Unique path type	Non-redundant path
C+C+	g-g-g- → tg-g- → tg-t → ttt → tg+t → tg+g+ → g+g+g+
	g-g-g- → g-g-t → tg-t → ttt → tg+t → g+g+t → g+g+g+
C+C-	g-g-g- → g-g-t → tg-t → ttt → tg+t → tg+g+ → g+g+g+
	g-g-g- → tg-g- → tg-t → ttt → tg+t → g+g+t → g+g+g+
T+C+	g-g-g- → tg-g- → tg-t → ttt → ttg+ → tg+g+ → g+g+g+
	g-g-g- → g-g-t → tg-t → ttt → g+tt → g+g+t → g+g+g+
	g-g-g- → g-g-t → g-tt → ttt → tg+t → g+g+t → g+g+g+
	g-g-g- → tg-g- → ttg- → ttt → tg+t → tg+g+ → g+g+g+
T+C-	g-g-g- → g-g-t → tg-t → ttt → ttg+ → tg+g+ → g+g+g+
	g-g-g- → tg-g- → tg-t → ttt → g+tt → g+g+t → g+g+g+
	g-g-g- → g-g-t → g-tt → ttt → tg+t → tg+g+ → g+g+g+
	g-g-g- → tg-g- → ttg- → ttt → tg+t → g+g+t → g+g+g+
C+M+	g-g-g- → tg-g- → tg-t → ttt → ttg+ → g+tg+ → g+g+g+
	g-g-g- → g-g-t → tg-t → ttt → g+tt → g+tg+ → g+g+g+
	g-g-g- → g-tg- → ttg- → ttt → tg+t → g+g+t → g+g+g+
	g-g-g- → g-tg- → g-tt → ttt → tg+t → tg+g+ → g+g+g+
C+M-	g-g-g- → g-g-t → tg-t → ttt → ttg+ → g+tg+ → g+g+g+
	g-g-g- → tg-g- → tg-t → ttt → g+tt → g+tg+ → g+g+g+
	g-g-g- → g-tg- → g-tt → ttt → tg+t → g+g+t → g+g+g+
	g-g-g- → g-tg- → ttg- → ttt → tg+t → tg+g+ → g+g+g+
T+T+	g-g-g- → tg-g- → ttg- → ttt → ttg+ → tg+g+ → g+g+g+
	g-g-g- → g-g-t → g-tt → ttt → g+tt → g+g+t → g+g+g+
T+T-	g-g-g- → tg-g- → ttg- → ttt → g+tt → g+g+t → g+g+g+
	g-g-g- → g-g-t → g-tt → ttt → ttg+ → tg+g+ → g+g+g+
T+M+	g-g-g- → g-g-t → g-tt → ttt → g+tt → g+tg+ → g+g+g+
	g-g-g- → tg-g- → ttg- → ttt → ttg+ → g+tg+ → g+g+g+
	g-g-g- → g-tg- → g-tt → ttt → g+tt → g+g+t → g+g+g+
	g-g-g- → g-tg- → ttg- → ttt → ttg+ → tg+g+ → g+g+g+
T+M-	g-g-g- → g-g-t → g-tt → ttt → ttg+ → g+tg+ → g+g+g+
	g-g-g- → tg-g- → ttg- → ttt → g+tt → g+tg+ → g+g+g+
	g-g-g- → g-tg- → g-tt → ttt → ttg+ → tg+g+ → g+g+g+
	g-g-g- → g-tg- → ttg- → ttt → g+tt → g+g+t → g+g+g+
M+M-	g-g-g- → g-tg- → g-tt → ttt → ttg+ → g+tg+ → g+g+g+
	g-g-g- → g-tg- → ttg- → ttt → g+tt → g+tg+ → g+g+g+
	g-g-g- → g-tg- → g-tt → ttt → g+tt → g+tg+ → g+g+g+
	g-g-g- → g-tg- → ttg- → ttt → ttg+ → g+tg+ → g+g+g+

**T+XT-**	g-g-g- → g-g-t → g-tt → g-tg+ → ttg+ → tg+g+ → g+g+g+
	g-g-g- → tg-g- → ttg- → g+tg- → g+tt → g+g+t → g+g+g+
**TXM**	g-g-g- → g-tg- → g-tt → g-tg+ → ttg+ → tg+g+ → g+g+g+
	g-g-g- → tg-g- → ttg- → g+tg- → g+tt → g+tg+ → g+g+g+
	g-g-g- → g-g-t → g-tt → g-tg+ → ttg+ → g+tg+ → g+g+g+
	g-g-g- → g-tg- → ttg- → g+tg- → g+tt → g+g+t → g+g+g+
**M+XM-**	g-g-g- → g-tg- → g-tt → g-tg+ → ttg+ → g+tg+ → g+g+g+
	g-g-g- → g-tg- → ttg- → g+tg- → g+tt → g+tg+ → g+g+g+

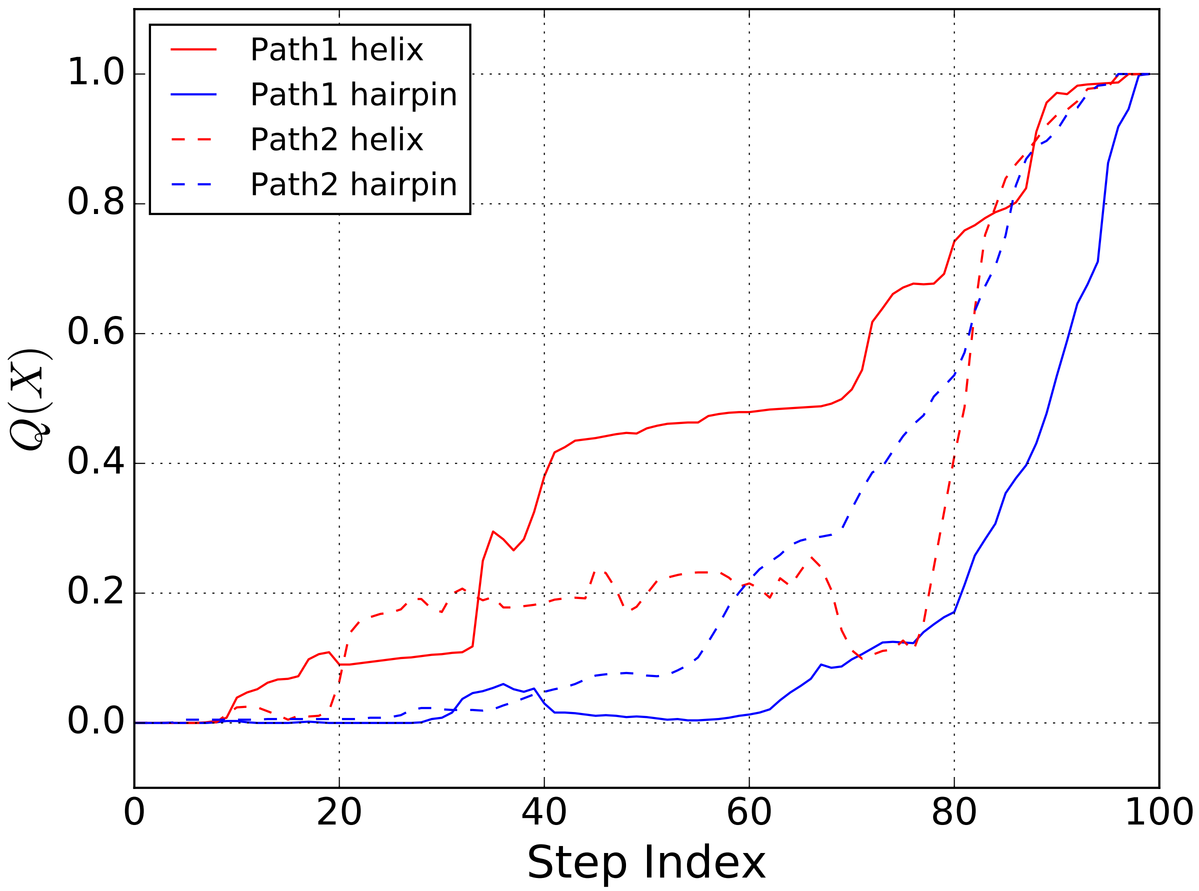

Supplementary Figure 1. A comparison of native contact fractions Q(x) of the helix and hairpin regions of FSD-1 along the lowest OM action pathways. *Red and blue lines represent the helix and the hairpin regions of FSD-1. Solid and dashed lines represent the lowest and second lowest OM action pathways.*
